# Identification of soil P fractions that are associated with P loss from surface runoff under various cropping systems and fertilizer rates on sloped farmland

**DOI:** 10.1371/journal.pone.0179275

**Published:** 2017-06-26

**Authors:** Xinghua Li, Baona Wang, Tewu Yang, Duanwei Zhu, Zhongnan Nie, Junchi Xu

**Affiliations:** 1 MOA Key Laboratory of Crop Ecophysiology and Farming System in the Middle Reaches of the Yangtze River, College of Plant Sciences and Technology, Huazhong Agricultural University, Wuhan, China; 2 The Cooperative Innovation Center for Sustainable Pig Production, Wuhan, China; 3 Laboratory of Plant Nutrition and Ecological Environment Research, Huazhong Agricultural University, Wuhan, China; 4 Department of Economic Development, Jobs, Transport and Resources, Hamilton, Victoria, Australia; University of Delhi, INDIA

## Abstract

Soil phosphorus (P) fractions and runoff P concentration were measured to understand the fate of soil P entering surface runoff water during summer cropping season of different double cropping systems under two fertilizer regimes. The dominant form of runoff P was particulate P (PP). Runoff total P (TP) was higher at the vegetative growth stage and lower at the crop reproductive stage. TP and PP were derived mainly from soil Olsen-P, Al-P and Fe-P and amounts increased with sediment content in runoff water. Runoff P discharge was closely related to the changes in soil P forms. Soil Olsen-P, mainly consisting of some Ca_2_-P and Al-P, was increased by elevating fertilizer rate. Along with crop growth, there were active interconversions among Olsen-P, Org-P, Fe-P and O-Al-P in the soil, and some available P converted into Ca_10_-P, with O-Fe-P possibly being a transitional form for this conversion. The oilseed rape/corn system had less runoff TP at the early stage, and wheat/sweet potato system had a lower runoff P at the late stage. Intercropping corn with sweet potato in the field with oilseed rape as a previous crop may be helpful for alleviating runoff P load during the summer in this region.

## Introduction

Phosphorus (P) enrichment has been recognized as the most critical stimulator to water eutrophication [1–3], and agriculture is considered a major source of P contamination in surface waters worldwide [4]. Where P is applied at rates exceeding crop uptake, it may built up in the soil and discharge into waterways through runoff during rainfalls [1, 5]. The identification of soil status that likely generates high runoff P concentrations is critical for developing rational P management strategies in agricultural systems [3]. Attempts have been made using agronomic soil test P (STP) to predict runoff P but the success is limited [6, 7]. They suggested that a measure of P buffering (PBI) may complement agronomic soil tests to improve the prediction [8, 9]. Dougherty et al. showed that either DGT (diffuse gradients in thin films) or Colwell P and PBI can be used to predict runoff P from six soils with a diverse range of soil P buffering properties and a wide range of P additions in repacked trays grown with pasture under simulated rainfall [3]. However, P concentration and forms in runoff usually vary considerably depending on soil properties, land use and management, fertilizer application, runoff delivery pathway, and other site-specific factors [9, 10], and the relationships between soil P and runoff P needs further study.

Phosphorus in soils exists in many complex chemical forms that differ markedly in their behavior, mobility, and resistance to bioavailability in the soils [11]. Therefore, the fate and transport of soil P vary largely depending on the forms [12]. Soil Olsen-P is considered available to plant [13–15] and therefore widely used to estimate soil P availability [16]; nevertheless, its association with P loss from runoff has not been clarified due to its varying components in different soils. Fractionation of soil P is not only an effective approach for investigating soil P availability and transformation [16, 17], but also can provide useful information for assessing the risk of soil P as the potential sources of eutrophication in aquatic systems [11]. However, it is very hard to identify individual compounds of soil P due to its complex chemistry [18]. The method to divide soil inorganic P (Pi) into various fractions was first developed by Chang and Jackson [13]. Further work on this method has led to a sequential Pi fractionation approach that has been widely used to divide soil Pi into L-P (NH_4_C1-extractable labile P), Al-P (NH_4_F-extractable Al phosphates), Fe-P (NaOH-extractable Fe phosphates), O-P (P occluded within oxides extracted with sodium citrate-Na_2_S_2_O_4_ solution) and Ca-P (H_2_SO_4_-extractable Ca phosphates) in acid and neutral soils [19]. However, the complex O-P should be sub-fractionated. In calcareous soils, the majority of Pi exists in various Ca-bound forms with different bioavailability to crops. A procedure was then developed for calcareous soil, which fractionates Ca-P into sub-fractions as Ca_2_-P [CaHPO_4_·nH_2_O], Ca_8_-P [Ca_8_H_2_(PO_4_)_6_·nH_2_O] and Ca_10_-P [Ca_10_(PO_4_)_6_·(OH)_2_] based on their availability and solubility [15, 20]. A complete P fractionation should also include organic P (Org-P) in soil. Because Ca-P fraction in non-calcareous soils is rather small [21], Lei et al. proposed a modified scheme, based on previous work, to fractionate soil P as Ca_2_-P, Al-P, Org-P, Fe-P, O-Al-P, O-Fe-P and Ca_10_-P [19]. Comparison studies indicated that the new scheme is applicable for acid and neutral soils, with higher P extraction rates than the methods of Chang and Jackson [13] and Gu and Jiang [20].

The response and availability of different soil P fractions to fertilizer application, crop types and rotation patterns has been studied in Northern China [17, 20] and Western Australia [22]. However, little is known about the transformation of soil P fractions under such agronomic practices and their association with P loss from farmlands. In this study, we have determined the differences in soil P fractions and runoff P concentration from various cropping systems over summer in a continuous double cropping experiment with different fertilizer regimes, which was conducted in Danjiangkou Reservoir region, Hubei Province, China. The major objective of this study was to establish a relationship between soil P fractions and runoff P loss for predicting P discharge from different cropping systems and clarify the transformation of soil P fractions in response to fertilizer application, crop types and rotation patterns.

## Materials and methods

### Ethics statement

The field experiment of our study did not involve any endangered or protected species and no specific permissions were required for our cropping experiments.

### Site description

A continuous double cropping experiment was conducted at Xijiadian (32°45’50”N, 111°9’42”E), Danjiangkou City, Hubei Province. The experimental site was about 5 km from Danjiangkou Reservoir that is the second largest artificial freshwater lake in Asia and also the headwater of the middle route of the South-to-North Water Transfer Project of China. From there, the water is transferred to major populous areas in northern China such as Beijing and Tianjin for drinking water. However, the water tends to be eutrophic with increasing total N and P concentrations in the reservoir [23] and the nutrient contamination has mainly come from soil erosion and agricultural activities [24]. The catchments surrounding the reservoir are typically rain-fed hilly farming regions and most dry fields are small (< 0.1 ha). Due to the shallow (< 20 cm in most slopping farmlands) and heavy-textured and poorly drained topsoil, the farmland is subject to severe soil erosion and high nutrient loss in the rainy seasons [25]. The surface runoff over the summer crop season accounted for over 70% of the total runoff of the entire cropping season [25]. The major crops grown on sloped upland are corn (*Zea mays* L.), sweet potato (*Ipomoea batatas* Lam.), winter wheat (*Triticum aestivum* L.) and oilseed rape (*Brassica napus* L.) with the major conventional double cropping systems as wheat and corn (wheat/corn), wheat and sweet potato (wheat/sweet potato) and oilseed rape and corn (rape/corn).

The climate is typical in the semi-humid northern subtropical zone, with average annual mean temperature of 15.9°C, average monthly mean temperature of 3.1°C (January) - 31.9°C (August) and frost-free period of 180–250 days. The long-term average annual precipitation is 834 mm with most of the rain falling in July to September.

The plot experiment was set up on a typically sloped farmland with a slope of 9.9° from October 2008 to October 2011. Sesame (*Sesamum indicum* L.) was grown without fertilizer application in the season prior to the experiment to control weeds and ensure the area was uniform for the experiment. The basic fertility and P fractions of the yellow brown clay were determined as described by Bao [26] and Lei et al. [19], respectively, after sesame harvest and before the commencement of the experiment. There were 5.17 g/kg of organic matter, 0.40 g/kg of total N, 9.54 g/kg of total K, 0.35 g/kg of total P, 33.87 mg/kg of alkali-hydrolysable N, 114.86 mg/kg of available K, 6.35 mg/kg of Olsen-P, 12.15 mg/kg of Ca_2_-P, 12.86 mg/kg of Al-P, 83.96 mg/kg of Fe-P, 19.71 mg/kg of O-Al-P, 197.21 mg/kg of Ca_10_-P and 12.96 mg/kg of Org-P in the soil. The pH and CaCO_3_ content of the soil was 6.97 and 3.5%, respectively.

### Experimental design and treatments

A factorial experiment with 6 treatments was arranged in a randomized complete block design (RCB) with 3 replicates. There were 3 continuous double cropping systems including winter oilseed rape/corn, wheat/corn and wheat/sweet potato under 2 fertilizer rates, i.e. a lower rate that was commonly used by farmers in the region, and a higher rate that mimicked potential fertilizer use in the future. The 3 cropping systems were combined with 2 fertilizer rates to form 6 factorial treatments: (1) rape/corn + lower fertilizer rate (RCL); (2) rape/corn + higher fertilizer rate (RCH); (3) wheat/corn + lower fertilizer rate (WCL); (4) wheat/corn + higher fertilizer rate (WCH); (5) wheat/sweet potato + lower fertilizer rate (WPL); (6) wheat/ sweet potato + higher fertilizer rate (WPH).

### Experimental infrastructure

There were 18 plots in total constructed in 3 rows in the field, each with 7.2 m in length, and 3.2 m in width (plot size = 23.0 m^2^). A concrete barrier about 15 cm wide was inserted 35 cm into bedrock and 25 cm above ground between individual plots to prevent lateral flow. A waterproof cement catchment grooves, which was at the same level as the topsoil on the top and 2 cm deep, was installed across the lower edge of each plot. One end of the catchment grooves was channeled to a water tank that had a total volume of 1 m^3^ and was covered to prevent rainfall entry and evaporation. A 55 L plastic bucket was placed in the tank to collect runoff water and sediment. The infrastructure was set up in the summer of 2008 and artificially adjusted soil slope of the plots after completion of construction.

### Crop establishment and harvest

All crops including winter crops were grown using the local conventional methods along the contour in plots. Oilseed rape (cv. Huaza 62) was sown with row spacing of 30 cm in the first ten days of October, and thinned out into 20 cm between plants at 5 leaf stage. Winter wheat (cv. Yumai 9203) was sown with row spacing of 25 cm in the second ten days of October. Corn (cv. Yanyu 18) was sown with row spacing of 60 cm in the last ten days of May to the first ten days of June, and thinned out into 30 cm between plants in a row at 4–5 leaf stage. Sweet potato (a local genotype) was transplanted in furrow-ridge tillage with a width of 40 cm (ridge height = 30 cm and furrow width = 30 cm). The distance between transplants was 30 cm on each ridge and the transplanting was undertaken in the last ten days of May up to mid June depending on rainfall. Oilseed rape and winter wheat was harvested in the middle and last ten days of May, respectively. Corn was harvested in the period from the end of September to the tenth of October, and sweet potato in the second ten days of October.

### Fertilizer application

The fertilizer applied for summer crops included 120.8 kg N/ha, 19.6 kg P/ha and 52.3 kg K/ha for the lower rate, and 169.1 kg N/ha, 27.5 kg P/ha and 73.2 kg K/ha for the higher rate; while, those for winter crops were 138.0 kg N/ha, 19.6 kg P/ha and 56.0 kg K/ha for the lower rate, and 193.5 kg N/ha, 27.5 kg P/ha and 78.4 kg K/ha for the higher rate. All phosphorus and potassium fertilizers were applied as base fertilizer prior to sowing both winter and summer crops. N fertilizer was split into 43% base fertilizer and 57% topdressing fertilizer for summer crops, and 64% base fertilizer and 36% topdressing fertilizer for winter crops. Topdressing N fertilizer was applied after thinning in late June for corn and 7 days after transplanting for sweet potato in summer crop season. In winter crop season, topdressing N fertilizer was applied at 5-leaf stage (12%) and bolting stage (24%) for oilseed rape and jointing stage for winter wheat. The fertilizers used in this study were urea (46% N), calcium superphosphate (12% P_2_O_5_) and potassium chloride (59% K_2_O). The rest of the management was based on the local conventional standard of field production.

### Data collection

In the present study, we focused on the summer cropping season in the fourth year (2011) of the experiment with high surface runoff in the entire cropping season and stabilized and uniformed soils under different treatments in plots. The amount of runoff water/sediment was recorded after rainfall events on 27 July and 23 August 2011, at the vegetative growth and reproductive stage of crops, respectively. All water samples were taken to the laboratory in iceboxes for determining the concentrations of sediment, total phosphorus (TP), particulate phosphorus (PP), total dissolved phosphorus (TDP), and dissolved inorganic P (DIP) in runoff. Three 4.8 cm diameter cores were taken to a depth of 20 cm from each plot and bulked to determine the concentration of Olsen-P, Org-P, Ca_2_-P, Al-P, Fe-P, O-Al-P, O-Fe-P and Ca_10_-P. Crop leaf area index (LAI) was measured at 3 random locations within a plot, using AccuPAR LP-80 (DECAGON DEVICES, INC.) at the vegetative growth (23 July) and reproductive stage (24 August), respectively. Crop economic organs (grain for corn and root tuber for sweet potato) and straw including corncob were sampled and oven-dried at 80°C to determine the dry yields and P contents after harvest.

### Measurement

Samples of runoff water were analyzed using the method of SEPA [27]. After the water was thoroughly mixed, a volume of 100 mL was sampled and then oven-dried at 105°C to determine sediment concentration. Phosphorus concentration in water was detected by molybdenum-antimony-ascorbic method [28]. Prior to determination, a blended water sample was digested with alkaline potassium persulfate under 120°C for 40 min for TP determination. The water sample after filtering through 0.45 μm pore diameter membrane was used to determine DIP. The filtered sample after being digested with alkaline potassium persulfate was used to determine TDP. The PP concentration was calculated by subtracting the TDP from TP; and the DOP (dissolved organic P) by subtracting the sum of DIP from TDP [29].

The dried plant samples were ground into fine powder and then digested with concentrated H_2_SO_4_ (98%, v/v) and HClO_4_ (70%, v/v) for determining the P content. Soil samples were air-dried and ground to pass a 150-μm mesh prior to analysis. Olsen-P was determined by the method of Olsen et al. [30]. Soil P fractionation was carried out using the sequential scheme of Lei et al. [19]. Phosphorus concentration in the digestion solutions of plant samples and in different extracts of the soils was determined by colourimetry [28].

### Statistical analysis

Data on the runoff amount, sediment and P concentrations, and the contents of soil Olsen-P and P fractions and crop LAI and P uptake of all treatments were analyzed using 2-way analysis of variance (ANOVA) with cropping systems in combination with different fertilizer rates as random factor and blocks (replicates) as fixed factor in a generalized linear model. Duncan’s multiple comparison test was subsequently used to determine significance of differences between treatments at *P* = 0.05. Pearson correlation and linear regression analysis was performed between Olsen-P and different P fractions in soil, and runoff TP and PP and soil Olsen-P and P fractions. All data analyses were performed using SPSS 17.0 (SPSS Inc., 2002).

## Results

### Crop leaf area index, yield and phosphorus uptake

**Fig 1:**
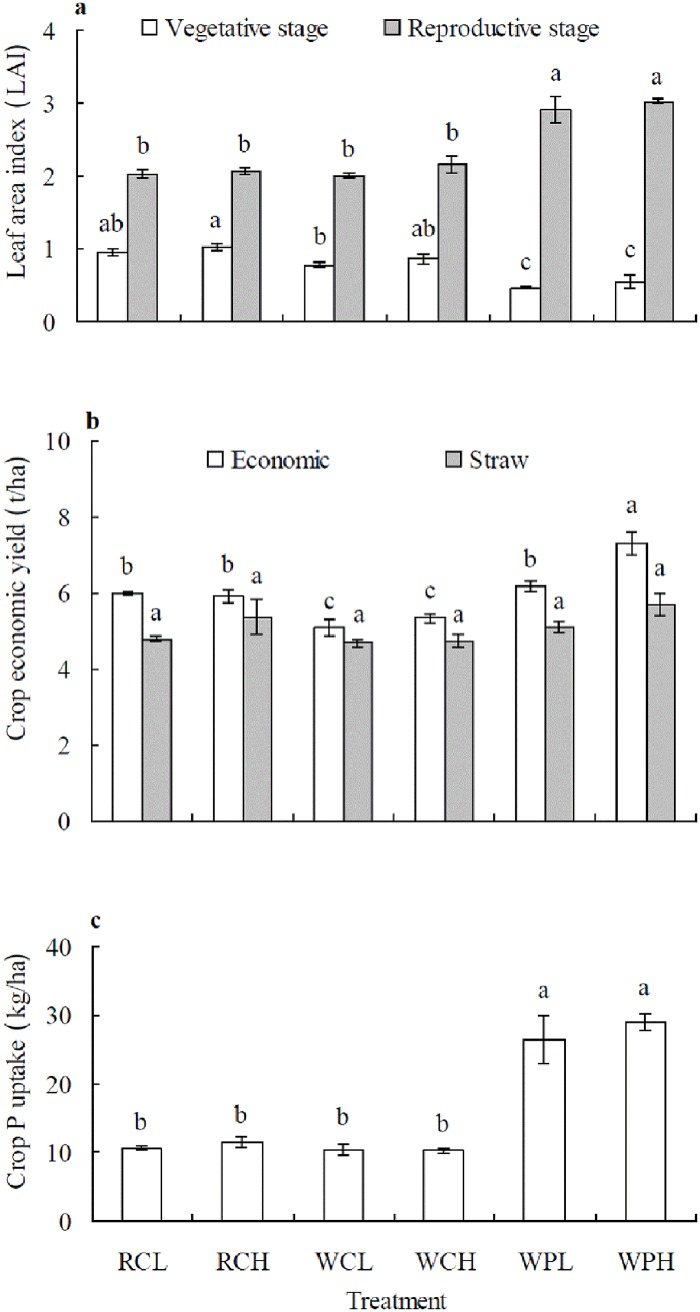
Crop leaf area index (LAI), yield and P uptake of summer crops in different cropping systems in the fourth year (2011). Data are means ± *SE*. Different letters in lower case above *SE* bars indicate significant differences between cropping systems at *P* < 0.05 at same stage. a: Leaf area index (LAI); b: Crop economic and straw yield; c: Crop P uptake. RCL: rape/corn + lower fertilizer rate; RCH: rape/corn + higher fertilizer rate; WCL: wheat/corn + lower fertilizer rate; WCH: wheat/corn + higher fertilizer rate; WPL: wheat/sweet potato + lower fertilizer rate; WPH: wheat/ sweet potato + higher fertilizer rate.

There were significant (*P* < 0.01) differences among different cropping systems in crop leaf area index (LAI) at vegetative growth and reproductive stages, economic yield and P uptake, but not in straw yield of summer crops (Fig 1). The LAI in wheat/sweet potato system under 2 fertilizer rates was lowest at the vegetative stage but highest at the reproductive stage. Elevating fertilizer rate tended to increase LAI but no significant difference detected between fertilizer application rates in the same cropping pattern (Fig 1a). The economic yield was highest for WPH in dry root tuber of sweet potato but lowest for WCL in dry corn grain. Elevating fertilizer rate increased economic yield in wheat/sweet potato. Corn yield was higher in rape/corn than in wheat/corn under 2 fertilizer rates (Fig 1b). More P was taken by summer crops in wheat/sweet potato with an increased P absorption rate of more than 129% than in other cropping systems. However, no significant differences were detected between fertilizer rates in same cropping patterns (Fig 1c).

### Runoff yield and sediment concentration

There was no significant difference in runoff yield among different cropping systems when measured at the vegetative stage with a rainfall of 24 mm. However, significant difference was detected in the sediment content of runoff, which was the highest for WCL but lowest for WPH (Table 1). Increasing fertilizer rate led to lower sediment content in all cropping patterns (Table 1). Contrasted to the vegetative stage, there was no significant difference in sediment content at reproductive stage when 18 mm of rainfall was received, while, significant difference in runoff yield was detected among cropping systems (Table 1). The runoff yield was highest for WPH, but lowest for RCL and RCH (Table 1).

**Table 1:** Runoff yield and sediment content in summer crop season in the fourth year (2011) of different cropping systems.

Treatment	Vegetative growth stage		Reproductive growth stage	
	Runoff yield (mm)	Sediment content (g/L)	Runoff yield (mm)	Sediment content (g/L)
RCL	0.9±0.2 a	3.7±0.6 b	0.1±0.0 c	0.5±0.0 a
RCH	0.9±0.1 a	1.1±0.4 c	0.1±0.0 c	0.4±0.2 a
WCL	1.0±0.2 a	6.8±0.2 a	0.4±0.1 ab	0.3±0.0 a
WCH	1.1±0.2 a	2.8±0.3 b	0.3±0.0 b	0.4±0.0 a
WPL	0.8±0.2 a	3.2±0.1 b	0.3±0.1 b	0.3±0.0 a
WPH	1.0±0.2 a	0.6±0.1 c	0.5±0.1 a	0.4±0.1 a
*F*_5, 12_	0.3814	16.734	8.306	0.221
*P*	0.852	<0.001	0.001	0.947

Data are Means ± *SE*. Means in a column followed by the same letter are not significantly different (α = 0.05) according to the Duncan’s Multiple Comparison test. ANOVA results are given in the lines F (F-value, numbers indicate the degrees of freedom) and *P* (level of significance).

### Phosphorus concentrations in runoff

**Fig 2:**
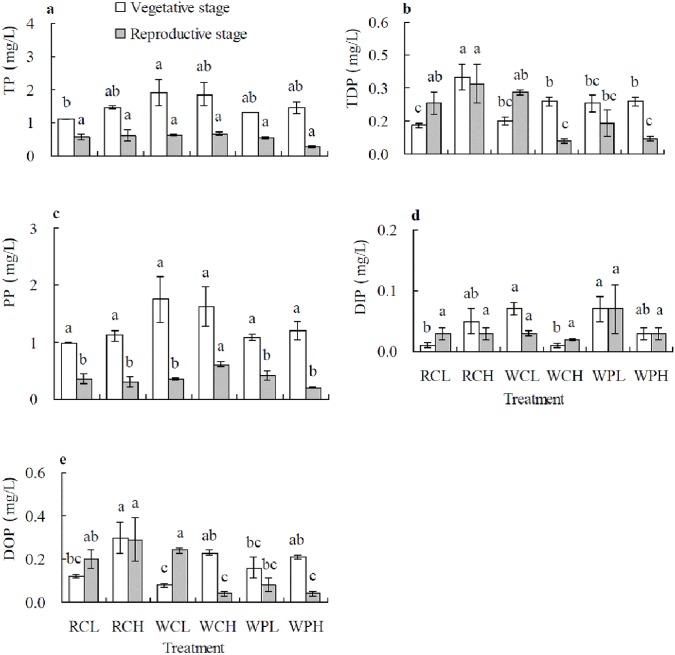
Concentrations of runoff P forms in different cropping systems at vegetative growth and reproductive stage of summer crops in the fourth year (2011). Data are means ± *SE*. Different letters in lower case above *SE* bars indicate significant differences between cropping systems at *P* < 0.05 at same stage. a: TP (total P); b: TDP (total dissolved P); c: PP (particulate P); d: DIP (dissolved inorganic P); e: DOP (dissolved organic P). RCL: rape/corn + lower fertilizer rate; RCH: rape/corn + higher fertilizer rate; WCL: wheat/corn + lower fertilizer rate; WCH: wheat/corn + higher fertilizer rate; WPL: wheat/sweet potato + lower fertilizer rate; WPH: wheat/ sweet potato + higher fertilizer rate.

Runoff P load varied substantially from different cropping systems. Significant (*P* < 0.05) differences were detected in the concentrations of runoff TP and DIP at the vegetative growth, PP at the reproductive stage, and TDP and DOP at both stages (Fig 2). Both TP and PP concentrations were higher at vegetative stage than at the reproductive stage in all cropping systems and PP accounted for most proportion of TP in the runoff (Fig 2a and 2c). The highest TP concentration was found in WCL, and highest TDP and DOP in RCH, but the lowest TP and TDP were found in RCL and lowest DOP in WCL, at the vegetative stage (Fig 2a, 2b and 2e). Runoff DIP concentration was rather small compared to other P forms. The higher DIP concentrations were found in WPL and WCL, but lower in RCL and WCH (Fig 2d). No differences were detected in PP among different cropping systems, although wheat/corn tended to have a higher PP under both higher and lower fertilizer application rates at the vegetative stage (Fig 2c). Increasing fertilizer rate led to increased TDP only in oilseed rape/corn and DOP both in oilseed rape/corn and wheat/corn, at the vegetative growth stage (Fig 2).

At the reproductive stage, there were no differences in runoff TP and DIP concentrations among cropping systems (Fig 2a and 2d). Runoff TDP and DOP were the highest in RCH, but lowest in WCH and WPH (Fig 2b and 2e). The WCH had a higher PP concentration than other systems (Fig 2c). Elevating fertilizer only increased PP concentration in wheat/corn (Fig 2c), however, resulted in lower TDP and DOP in wheat/corn (Fig 2b and 2e), at the reproductive stage.

### Soil Olsen-P and P fractions

**Fig 3:**
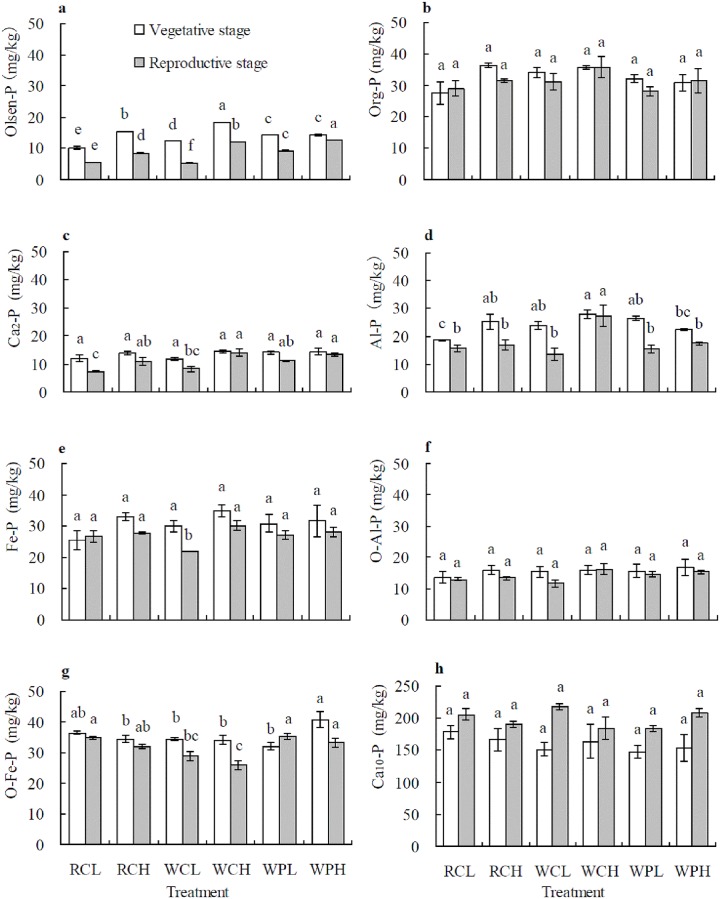
Soil Olsen-P and P fractions in different cropping systems at vegetative growth and reproductive stage of summer crops in the fourth year (2011). Data are means ± *SE*. Different letters in lower case above *SE* bars indicate significant differences between cropping systems at *P* < 0.05 at same stage. a: Olsen-P; b: Org-P; c: Ca_2_-P; d: Al-P; e: Fe-P; f: O-Al-P; g: O-Fe-P; h: Ca_10_-P. RCL: rape/corn + lower fertilizer rate; RCH: rape/corn + higher fertilizer rate; WCL: wheat/corn + lower fertilizer rate; WCH: wheat/corn + higher fertilizer rate; WPL: wheat/sweet potato + lower fertilizer rate; WPH: wheat/ sweet potato + higher fertilizer rate.

The contents of soil Olsen-P, Ca_2_-P and Al-P were relatively higher at the vegetative stage than at reproductive stage for all cropping systems. This was the case for Org-P, Fe-P, O-Al-P and O-Fe-P in most cropping systems. However, Ca_10_-P content increased to different degrees at the reproductive stage compared to vegetative stage in all cropping systems (Fig 3). No difference was detected in the content of soil Ca_2_-P and Fe-P (Fig 3c and 3e) at the vegetative stage, and Org-P, O-Al-P and Ca_10_-P (Fig 3b, 3f and 3h) at both stages among cropping systems. At the vegetative stage, there was a trend for soil Olsen-P content as: WCH > RCH > WPH > WPL > WCL > RCL (Fig 3a); for Al-P as: WCH > WPL > RCH > WCL > WPH > RCL (Fig 3d); and for O-Fe-P as: WPH > RCL > RCH > WCL > WCH > WPL (Fig 3g). Elevating fertilizer rate led to increases of soil Olsen-P in oilseed rape/corn and wheat/corn patterns (Fig 3a), Al-P in oilseed rape/corn and O-Fe-P in wheat/sweet potato (Fig 3g) at the vegetative stage.

At the reproductive stage, there was a trend for soil Olsen-P as: WPH > WCH > WPL > RCH > RCL > WCL (Fig 3a); for Ca_2_-P as: WCH > WPH > WPL > RCH > WCL > RCL (Fig 3c); and for O-Fe-P as: WPL > RCL > WPH > RCH > WCL > WCH (Fig 3g). Soil Al-P content was significantly higher for WCH than for other cropping systems (Fig 3d). Soil Fe-P content was significantly lower for WCL than for other cropping systems (Fig 3e). Elevating fertilizer rate increased soil Olsen-P in all cropping systems (Fig 3a), Ca_2_-P in oilseed rape/corn and wheat/corn (Fig 3b), Al-P and Fe-P in wheat/corn (Fig 3d and 3e).

### Relationship of soil Olsen-P, Ca_10_-P and runoff P with soil P fractions

Table 2 shows that soil Olsen-P was significantly positively correlated with Org-P, Ca_2_-P, Al-P, Fe-P and O-Al-P but negatively with Ca_10_-P. Contrasted to Olsen-P, soil Ca_10_-P was significantly negatively correlated with Ca_2_-P, Al-P, Fe-P and O-Al-P (Table 2).

**Table 2:** Correlation coefficients between soil Olsen-P and Ca_10_-P and other P fractions in summer crop season of different cropping systems.

	Olsen-P	Org-P	Ca_2_-P	Al-P	Fe-P	O-Al-P	O-Fe-P	Ca_10_-P
Olsen-P	1.000	0.639*	0.929**	0.851**	0.908**	0.877**	0.239	-0.730**
Ca_10_-P	-0.730**	-0.364	-0.664*	-0.764**	-0.732**	-0.716**	-0.400	1.000

* and ** indicate significance at *P* < 0.05 or 0.01, respectively.

**Fig 4:**
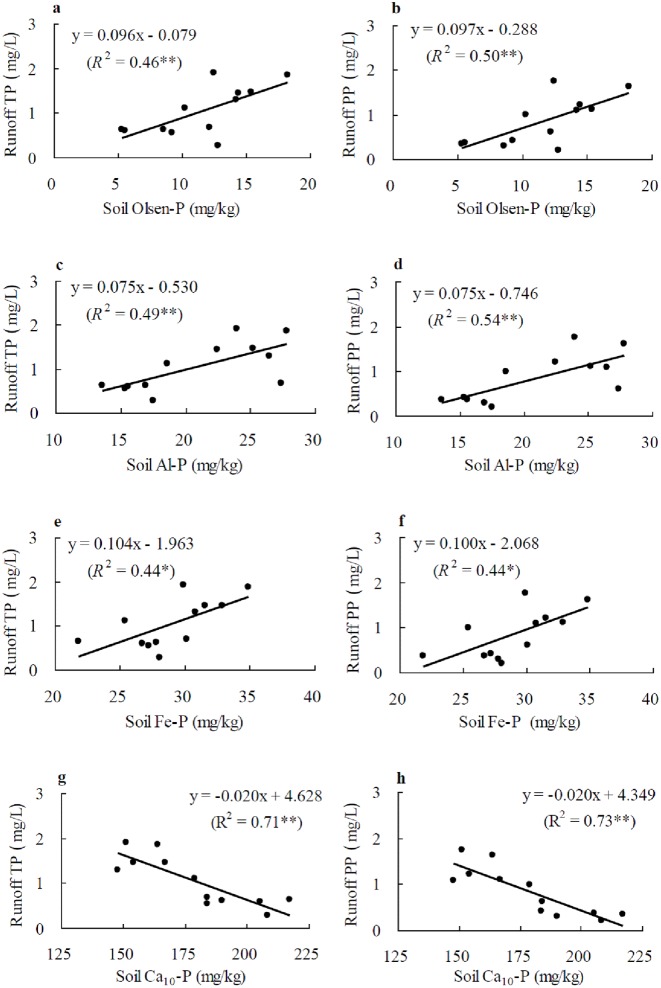
Linear regression of runoff TP and PP with soil Olsen-P and P fractions. a: runoff TP with soil Olsen-P; b: runoff PP with soil Olsen-P; c: runoff TP with soil Al-P; d: runoff PP with soil Al-P; e: runoff TP with soil Fe-P; f: runoff PP with soil Fe-P; g: runoff TP with soil Ca_10_-P; h: runoff PP with soil Ca_10_-P. * and ** indicate significance at *P*<0.05 and 0.01, respectively.

Regression analysis revealed that both runoff TP and PP were positively (*P* < 0.05 or 0.01) associated with soil Olsen-P (*R*^2^ = 0.46 for TP and 0.50 for PP), Al-P (*R*^2^ = 0.49 for TP and 0.54 for PP) and Fe-P (*R*^2^ = 0.44 for both TP and PP), but negatively (*P* < 0.01) with Ca_10_-P (*R*^2^ = 0.71 for TP and 0.73 for PP) (Fig 4).

## Discussion

The relative importance of surface and subsurface flows for pollutant transport has been studied at plot scale [31, 32] and the surface flow identified as an important factor leading to soil P loss from sloped farmland into waterways [2, 33]. The dominant P form in the runoff water was PP in this study, which is in agreement with the previous findings in a simulated rainfall experiment [34]. The P load in surface flows is mainly derived from desorption in surface soils [35, 36] and P loss of overland flow was mainly through soil transport [37]. The primary form of dissolved P was organic P, although it contributed only small amount to runoff TP in this study. This may be attributed to the higher mobility of organic P due to lower fixation by soil inorganic minerals compared to inorganic P [38]. Leytem et al. [39] and Pagliari and Laboski [40] also reported that the mobility of soil P can be increased by manure application. Phosphorus loss via surface runoff varies spatially and temporarily depending on the magnitude and intensity of rainfall and site specificities such as vegetation and soil materials [41]. The concentrations of TP and PP as well as sediment in the runoff water were much smaller at the reproductive stage than at vegetative growth in all cropping systems of this study. This is partially ascribed to the higher quantity and intensity of rainfall that was received at the vegetative stage. TP and PP concentrations are also related to crop growth. Plant coverage became greater as shown in LAI measurement at the reproductive stage, which could reduce the kinetic energy of raindrops so as to prevent surface soil erosion [42]. The growth of root systems may also have played an important role in the control of P runoff loss, because they could contribute to soil compaction [43, 44], and soil structural stability [45], therefore, increase the soil infiltration capacity [46]. McDowell et al. [7] and Shigaki et al. [47] also showed that applied fertilizer P is likely released during the initial storms.

Numerous studies have shown that runoff P concentration is highly related to soil P status and P application [5, 6, 8, 48]. When P is applied to soil, it undergoes a complex interconversion among different forms or fractions through chemical, physical and biological processes [17, 38]. In general inorganic P (Pi) dominates the soil P pools with different fractions [17, 38]. In this study, soil Pi presented mainly in Ca_10_-P. Higher fertilizer rate tended to increase Olsen-P as well as Ca_2_-P in all cases and Al-P, Fe-P, O-Al-P and Org-P in most cases, compared to the lower fertilizer application rate. From the vegetative growth to reproductive stage, there was a consistent decrease in soil Olsen-P, Ca_2_-P and Al-P but an increase in Ca_10_-P. Nevertheless, Fe-P, O-Al-P and O-Fe-P decreased only by relatively smaller amounts or even increased in some cases. From this and together with the correlation analysis, it was concluded that Olsen-P was mainly from Ca_2_-P and partially from Al-P and there were active interconversions among Olsen-P, Org-P, Fe-P and O-Al-P, and some Olsen-P was converted into Ca_10_-P along with crop growth. Apart from the effects of crop growth and P uptake, Ca_10_-P formation contributes to reduced P load in runoff flow because of its immobility; however, this may result in low soil P available to plant [49]. Meanwhile, Olsen-P had little correlation with O-Fe-P, which implies that O-Fe-P may be a major transitional form of available P to Ca_10_-P.

Our study showed that runoff TP and PP were derived mainly from Olsen-P, Al-P and Fe-P in the soil. Yu et al. indicated that Ca_2_-P showed the largest availability to plant followed by Al-P and Fe-P in different soil Pi fractions [50]. This indicated that the availability of soil Pi fractions also contributes to P runoff loss. However, Ca_2_-P, Org-P and O-Al-P were insignificantly related to runoff P loss, although they could convert to Olsen-P. Soil Ca_2_-P is the Pi fraction preferred by crops [50], which might explain why it is not closely related to P concentration in runoff water. While Org-P content was relatively lower and accounted only for a small proportion in soil P pool in this study, O-Al-P was less mobile compared to other available Pi fractions. In this regard, O-Al-P formation may be conducive to reducing runoff P discharge while supplying P to crops [49].

In this study, higher fertilizer rate did not significantly increase the runoff TP in cropping systems. Phosphorus was applied as basal fertilizer, and the nutrients in the fertilizer applied not only included P but N and K. These might have stimulated plant growth with relatively more P uptake, leading to less loss of the nutrient as crops grew. Runoff increased and TP concentration tended to decrease at the reproductive stage under the higher fertilizer rate for wheat/sweet potato, compared to the other cropping systems. In addition to the dilution of P by more runoff water, this was also related to the characteristics of the creeping growth behavior of sweet potato. When sweet potato branches, the ground coverage increases rapidly, the progress of which is enhanced by higher fertilizer rate. This may have reduced infiltration velocity of rain water [51], leading to more surface runoff and decrease in PP due to the filtering effects as vegetation coverage increases [52]. Runoff TP was lower in oilseed rape/corn than in wheat/corn at the vegetative stage, probably due to more P removed from the soil through higher corn yield in the oilseed rape/corn pattern. In this regard, intercropping corn with sweet potato in the field with oilseed rape as a previous crop may be helpful for alleviating runoff P load during the summer in this region. In oilseed rape/corn, however, runoff DOP was relatively higher under the higher fertilizer rate than in other cropping systems for both vegetative and reproductive stages. The possible reason was that oilseed rape is a deciduous species from which almost all leaves fall before harvest, and those leaves may have increased the organic matter in soil, particularly under the higher fertilizer rate. However, DOP generally contributed only a small part to TP in this study; therefore, the effect of DOP runoff from different cropping systems needs further investigation. Moreover, the linear regressions of runoff TP and PP with soil Olsen-P, Al-P and Fe-P fractions showed relatively smaller *R*^2^ values in this study, although all regression analyses are significant. This may be attributed to the methods used to measure P in soils and runoff water in this study. Do Nascimento et al. [53] recently reported that digestion and colorimetric methods may affect the precise determination of TP and organic P in samples and suggested using ICP-OES to measure the undigested extracts in sequentially fractionated soil. In addition, we only investigated soil P status and runoff P at two representative stages over the summer in this study. Further studies using improved methodology are needed to understand the relationship between the changes in soil P fractions and their effects on P runoff throughout the entire growing season.

Sharpley et al. reported that runoff water is considered as degraded if its total P exceeds a guideline concentration of 0.10 mg P/L [4]. Total P concentration in the runoff water varied in different cropping systems but was far beyond this threshold both at the vegetative and reproductive stages of the summer crops in this study. Therefore, controlling P runoff from farmland is urgently required to protect the water quality of Danjiangkou Reservoir. Our study was only performed at a plot scale and a field-scale study is needed.

## Conclusion

There were differences in runoff P loss in the summer crop season under different cropping systems and fertilizer rates. Runoff TP concentration was relatively higher at the vegetative stage but decreased at the reproductive stage for all cropping systems. Particulate P was the major form in runoff. Higher runoff sediment yields resulted in higher runoff TP and PP. Runoff TP and PP were mainly derived from soil Olsen-P, Al-P and Fe-P. Soil Olsen-P that was mainly consisted of some Ca_2_-P and Al-P was increased by elevating fertilizer rate. Along with crop growth, there were active interconversions among Olsen-P, Org-P, Fe-P and O-Al-P in the soil, and some available P changed to Ca_10_-P, with O-Fe-P as a transitional form. Runoff TP was lower at the early stage in oilseed rape/corn and at the late stage in wheat/sweet potato. Intercropping corn with sweet potato following oilseed rape may help reduce runoff P load during the summer in this region.

## Supporting information

S1 File Original crop leaf area index (LAI), yield and P uptake data. RCL: rape/corn + lower fertilizer rate; RCH: rape/corn + higher fertilizer rate; WCL: wheat/corn + lower fertilizer rate; WCH: wheat/corn + higher fertilizer rate; WPL: wheat/sweet potato + lower fertilizer rate; WPH: wheat/ sweet potato + higher fertilizer rate.
(XLS)

S2 File Original runoff yield and sediment content data. RCL: rape/corn + lower fertilizer rate; RCH: rape/corn + higher fertilizer rate; WCL: wheat/corn + lower fertilizer rate; WCH: wheat/corn + higher fertilizer rate; WPL: wheat/sweet potato + lower fertilizer rate; WPH: wheat/ sweet potato + higher fertilizer rate.
(XLS)

S3 File Original runoff P forms concentration data. RCL: rape/corn + lower fertilizer rate; RCH: rape/corn + higher fertilizer rate; WCL: wheat/corn + lower fertilizer rate; WCH: wheat/corn + higher fertilizer rate; WPL: wheat/sweet potato + lower fertilizer rate; WPH: wheat/ sweet potato + higher fertilizer rate.
(XLS)

S4 File Original soil Olsen-P and P fractions content data. RCL: rape/corn + lower fertilizer rate; RCH: rape/corn + higher fertilizer rate; WCL: wheat/corn + lower fertilizer rate; WCH: wheat/corn + higher fertilizer rate; WPL: wheat/sweet potato + lower fertilizer rate; WPH: wheat/ sweet potato + higher fertilizer rate.
(XLS)
