# Correction: Identification of soil P fractions that are associated with P loss from surface runoff under various cropping systems and fertilizer rates on sloped farmland

**DOI:** 10.1371/journal.pone.0191753

**Published:** 2018-01-19

**Authors:** Xinghua Li, Baona Wang, Tewu Yang, Duanwei Zhu, Zhongnan Nie, Junchi Xu

In the Funding and Acknowledgements section, the grant number from the funder National Key Research and Development Program of China is listed incorrectly. The correct grant number is: 2017YFD0800102.
